# High rates of homicide in a rural South African population (2000–2008): findings from a population-based cohort study

**DOI:** 10.1186/s12963-015-0054-0

**Published:** 2015-08-07

**Authors:** George Otieno, Edmore Marinda, Till Bärnighausen, Frank Tanser

**Affiliations:** Kenya Medical Research Institute, P.O Box 1578, 40100 Kisumu, Kenya; Centre for Disease Control and Prevention, Atlanta, GA USA; Wellcome Trust Africa Centre for Health and Population Studies, P.O Box 198, Mtubatuba, South Africa; Department of Global Health and Population, Harvard School of Public Health, Boston, USA; School of Public Health, University of Witwatersrand, 7 York Road, Parktown, 2193 Johannesburg, South Africa

## Abstract

**Background:**

South Africa has continued to receive increasing attention due to unprecedented high levels of violence. Homicide-related violence accounts for a significant proportion of unnatural deaths and contributes significantly to loss of years of expected life. We investigated levels and factors associated with homicide-related deaths and identify communities with excessively high homicide risk in a typical rural South African population.

**Method:**

Data drawn from verbal autopsies conducted on all deaths recorded during annual demographic and health surveillance in KwaZulu Natal, South Africa were used to derive the cumulative probability of death from homicide over a nine-year period (2000–2008). Weibull regression methods were used to investigate factors associated with homicide deaths. A Kulldorff spatial scan statistic was used to identify spatial clusters of homicide-related deaths.

**Results:**

With 536 homicide-related deaths, and a median seven years of follow-up, the study found an overall homicide incidence rate of 66 deaths per 100, 000 person-years of observation (PYOs) (95 % CI 60-72) for the period under study. Death related to the use of firearms was the leading reported method of homicide (65 %) and most deaths occurred over weekends (43 %). Homicides are the second-most common cause of death in men aged 25–34 after HIV-related deaths (including TB) in this community, at 210 deaths per 100,000 PYOs, and was highest among 55–64 year old women, at 78 deaths per 100,000 PYOs. Residency status, age, socioeconomic status, and highest education level attained independently predicted the risk of homicide death. The spatial distribution of homicide deaths was not homogenous and the study identified two clear geographical clusters with significantly elevated homicide risk.

**Conclusion:**

The high rates of homicide observed in this typical rural South African population – particularly among men – underscore the need for urgent interventions to reduce this tragic and theoretically preventable loss of life in this population and similar South African settings.

## Introduction

Globally, violence has been recognized as one of the leading public health issues [1, 2]. No country or community is untouched by violence, with over 1.4 million people worldwide losing their lives to violence every year (WHO report 2013) [3]. Homicide is an extreme form of violence, contributing to loss of years of expected life within a society or a country. More than 20 years after the end of apartheid, South Africa continues to experience excessive levels of violence [4, 5]. Homicide accounts for 56 % of fatal injuries among individuals aged 15–34 years [5, 6]. Although South Africa’s homicide rate may be dropping slowly, it is still among the world’s highest outside a war zone [4]. The 2013 Global Study on Homicide report indicates that Southern Africa and Central America have homicide rates over four times higher than the current global average rate of 6.2 per 100, 000 population, making them the subregions with the highest homicide rates on record [7].

According to the Johannesburg-Based Centre for the Study of Violence and Reconciliation, South Africa’s current high rate of violent crime is related to social and economic marginalization of a large section of the population, which stemmed from the apartheid system. Poverty and inequality are likely to have contributed to South Africa’s burden of violent injury deaths [4, 5]. Coovadia et al describe how apartheid and colonial policies were used to generate great wealth for select racial groups while most of the population lived in abject poverty [8]. This inequality is hypothesized to have fueled aggressive behavior as a reaction to social bias and discrimination, which resulted in an increase in violent crime.

South Africa has an established system for registration of births, deaths, and causes of death through the vital registration systems and National Injury Mortality Surveillance Systems (NIMSS). While civil registration systems remain underdeveloped in most African countries [9], recent political and public service transformations in South Africa have focused on improving coverage of civil registration systems to meet the international standards [10], and as such, South African cause-specific mortality patterns have been used to model mortality in many sub-Saharan Africa countries [10]. However, despite this improvement, South Africa still assigns many injury deaths to ICD codes that are indeterminate with regard to intent, although it did bridge coding in 1996 using ICD-9 and ICD-10 at the three character level. However, statistics are reported only according to ICD-9 [10, 11], and thus the incidence of many important mechanisms of injuries cannot be determined. Usually the key dump codes (e.g., ICD-10 code: X59) are grouped together with other specified mechanisms and without access to data on these injury dump codes, and the quality of the data overall cannot be assessed and injury mortality cannot be reliably estimated [12]. The Africa Centre Demographic Information System (ACDIS) provided a unique opportunity to study homicide in a complete rural population using verbal autopsy data collected routinely. We investigated the levels and factors associated with homicide-related deaths, and documented the subgroups and geographical areas of high risk in a rural culturally homogeneous population of KwaZulu-Natal (KZN), South Africa.

## Methods

### Study area

The Africa Centre demographic surveillance area (DSA), in the Umkhanyakude district of northern KZN, covers 438 km^2^ in size and includes deep rural areas, a township and peri-urban informal settlements. The study longitudinally follows a population cohort of approximately 87,000 persons in 11,538 households. The population is predominantly Zulu. Although it is a largely rural area, subsistence agriculture is not common; the principal source of income for most households is waged employment and state pension. The majority of the people are poor; only two in five adults are formally employed. Literacy levels, access to electricity and clean water were 78 %, 51 %, and 62 %, respectively, in 2011 [13]. Life expectancy has been severely affected by the HIV pandemic but has increased markedly in the last decade due to the rapid scale up in ART [14]. In 2003, the year before ART became available in the public-sector health system, adult life expectancy was 49.2 years; by 2011, adult life expectancy had increased to 60.5 years—an 11.3-year gain [15]. Unlike many others parts of Africa where homesteads are clustered in clearly identifiable villages, most of the rural population in KwaZulu-Natal lives in scattered multigenerational homesteads of varying sizes (1–100) [16]. The area also experiences substantial circulatory in- and out-migration [17].

Routine surveillance visits to each household were conducted three times a year, from 2000 to 2003 and twice a year thereafter. At each visit, a questionnaire was administered to key household informants providing up-to-date information on all household members; those residing in the DSA and those that are considered nonresidents. Demographic and health information was collected prospectively every six months from all registered households. Household socio-economic status, employment, and education data were collected once a year.

Ethical approval for the ACDIS research was obtained from the Research Ethics Committee of the Nelson R. Mandela School of Medicine, University of Natal. The title of the approved protocol is: ‘A Socio-demographic platform for population-based reproductive health research in a rural district of KwaZulu-Natal’ (Ref E009/00, September 2000). Annually, this protocol has to be recertified. For any changes to that protocol or development of new modules, separate protocols are submitted to the Research Ethics Committee as an amendment to the approved protocol.

### Measurement of homicide

ACDIS collects longitudinal information on residents as well as nonresidents who retain membership in household in the surveillance area. Residents reside at a physical structure within the surveillance area at a particular point in time, whereas nonresident members (self-defined by members of the household) retain links to the household but are not physically present in the surveillance area. A partial resident is a person who was not resident in the surveillance area for the entire period of observation. Verbal autopsies [18] are used to provide data on illness history prior to death and possible causes of death [19]. All registered individuals, both residents and nonresidents as of January 1, 2000 to December 31, 2008, were included in this study (*N* = 126, 462, 814,715 person-years of observation).

Verbal autopsy interviews with a close caregiver of the deceased were conducted for every death by a trained nurse. The nurse used a validated verbal autopsy questionnaire [19]. Two physicians, using the collected data, disease history, signs, and symptoms, independently assign probable cause of death. Where there were differences in assigned cause of death, a third physician blinded to the other results reviewed the verbal autopsy information and assigned a cause. If two of the three results agreed, a consensus was reached among the three coders and a decision made, otherwise the cause of death assigned as undefined. Validation studies have shown good overall sensitivity and specificity in comparison with physician’s certification [20, 21]; specificity and sensitivity of intentional injuries has been measured at 70 % and 98 %, respectively [20]. Homicide was defined as the intentional killing of a human being by another person or criminal negligence that causes the death of another person.

### Statistical analysis

Data were analyzed using Stata version 11.0 (Corporation, College Station, Texas, USA). Cumulative probabilities of homicide were estimated using Kaplan Meier (K-M) methods. Follow-up started on January 1, 2000, and observations were censored on the December 31, 2008, or the last time the person was resident in the demographic site. Homicide incidence rates were computed using number of deaths as the numerator and person-time of follow-up as the denominator. We performed a Mann-Whitney rank-sum test for trends in homicide rate over time. Univariate and multivariate Weibull parametric regression models were fitted to investigate factors associated with homicide. Unlike the Cox regression model, a Weibull model allows the hazards functions to either increase or decrease monotonically over time. Categorical variables with missing data were coded ‘unknown’ to maintain the overall denominator (Socio-economic (10 %), Age (0.8 %)). A household asset was created using an assets count, following summation using the method of Case et al [22]. Briefly, household items were combined into a single aggregate using Principal Component Analysis, ranked using index, and divided into quintiles from poorest (“1”) , very poor (“2”), poor (“3”), less poor (“4”), and least poor (“5”), representing the lowest socio-economic position to the highest socio-economic position, respectively.

### Spatial analysis

ACDIS has developed and maintained a Geographical Information System (GIS) that allows the detailed spatial analysis of all data collected. All homesteads and facilities in the area have been mapped by field workers using differential global positioning systems. To identify clusters of homicide and test the null hypothesis of uniform distribution within the DSA, all mortality events and person-years of observation for all those resident in the surveillance area at any stage during the period of observation (a total of 304,047 person-years of observation) were summed at each of the 11,000 homesteads in the study area. We then applied Kulldorff’s spatial scan statistic implemented in SaTScan software version 8.0 to perform spatial analysis scanning to detect any clustering of homicide events across the surveillance area [23]. The analysis was done using SaTScan spatial cluster detection program [23, 24]. A purely spatial distribution scan statistic using the exponential probability model, which is designed for survival data, was employed. A spatial scan statistic is a cluster detection test that is able to both detect the location of cluster and evaluate their statistical significance without the problems associated with multiple testing. A detailed description of the methodology is given elsewhere [24]. Briefly, the spatial scan statistic imposes a circular window on a map, and it allows the center of the circle to move across the study region. For any given position of the center, the radius of the circle changes continuously so that it can take any value from zero up to a specified maximum value. For each potential cluster, a likelihood ratio test statistic was used to determine if the number of homicide cases within the potential cluster was higher than expected. Expected numbers of homicide cases were calculated on the basis of the null hypothesis of complete spatial randomness. We allowed the clusters to overlap by <50 % and set the maximum search radius of the circle to be 3 km. The advantage of such an approach is that we do not aggregate the homicide data by arbitrary administrative units (resulting in loss of spatial precision) but instead use the precise spatial location of each individual to identify the clusters using the Kulldorff spatial scan statistic.

## Results

**Fig. 1:**
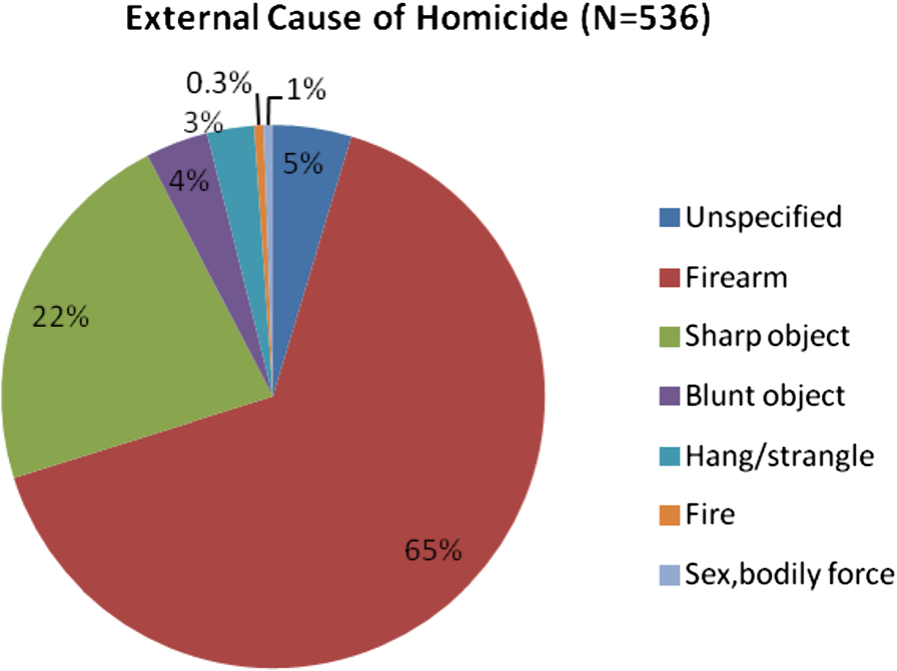
External causes of death (2000–2008)

**Fig. 2:**
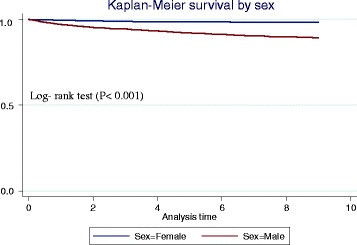
Homicide-free survival among men and women (2000–2008)

A total of 126,462 individuals were included in the study, and 43 % were male (Table 1). The majority of the population resided in rural areas (52 %). A total of 536 homicide-related deaths were recorded between 2000 and 2008. Death by firearms took the lead by 65 % with hand guns accounting for 53 % of these deaths (Fig. 1). Most deaths were reported on a Saturday (21 %). Males had a higher risk of homicide deaths in comparison to females as shown by the Kaplan-Meier curves on cumulative probabilities of homicide deaths (Fig. 2); Log- rank test (P < 0.001).

**Table 1:** Univariate Weibull regression analysis of homicide risk in a rural setting, KwaZulu-Natal (2000–2008)

	Female		Male	
Individual risk factor	Rate	HR (95 % CI)	Rate	HR (95 % CI)
Residency				
Resident	28.88		116.84	
Partial-resident	10.43	0.36 (0.21, 0.62)	80.75	0.68 (0.54, 0.85)
Nonresident	12.21	1.62 (0.92, 2.87)	301.33	2.58 (2.07, 3.22)
Education				
None	19.92		57.69	
Primary	3.86	1.07 (0.58, 1.99)	111.37	1.92 (1.34, 2.76)
Post-primary	25.01	1.23 (0.68, 2.22)	163	2. 92 (2.06, 4.12)
Household Socio-economic status				
Poorest	25.68		81.07	
Very poor	22.84	0.88 (0.38, 2.05)	105.33	1.30 (0.80, 2.09)
Poor	18.32	0.71 (0.30, 1.69)	104.57	1.29 (0.81, 2.04))
Less poor	22.73	0.88 (0.39, 1.97)	153.68	1.89 (1.23, 2.91)
Least poor	19.14	0.74 (0.31, 1.76)	111.69	1.37 (0.87, 2.18)
Age group (years)				
15–24	18.3		106.34	
0–14	4.49	0.24 (0.08, 0.71)	1.93	0.03 (0.01, 0.10)
25–34	6.262	1.44 (0.77, 2.71)	223.2	2. 10 (1.64, 2. 69)
35–44	16.65	0.90 (0.39, 2.07)	220.78	2.08 (1.57, 2.77)
45–54	23.72	1.29 (0.56, 2.97)	164.56	1.55 (1.09, 2.20)
55–64	75.51	4.13 (2.05, 8.31)	142.67	1.34 (0.84, 2.15)
65+	61.14	3.33 (1.72, 6.47)	134.18	1.27 (0.77, 2.07)

A household socio-economic status was collected among residents only ~ 10 % missing information; homicide rate per 100,000 PYOs

**Fig. 3:**
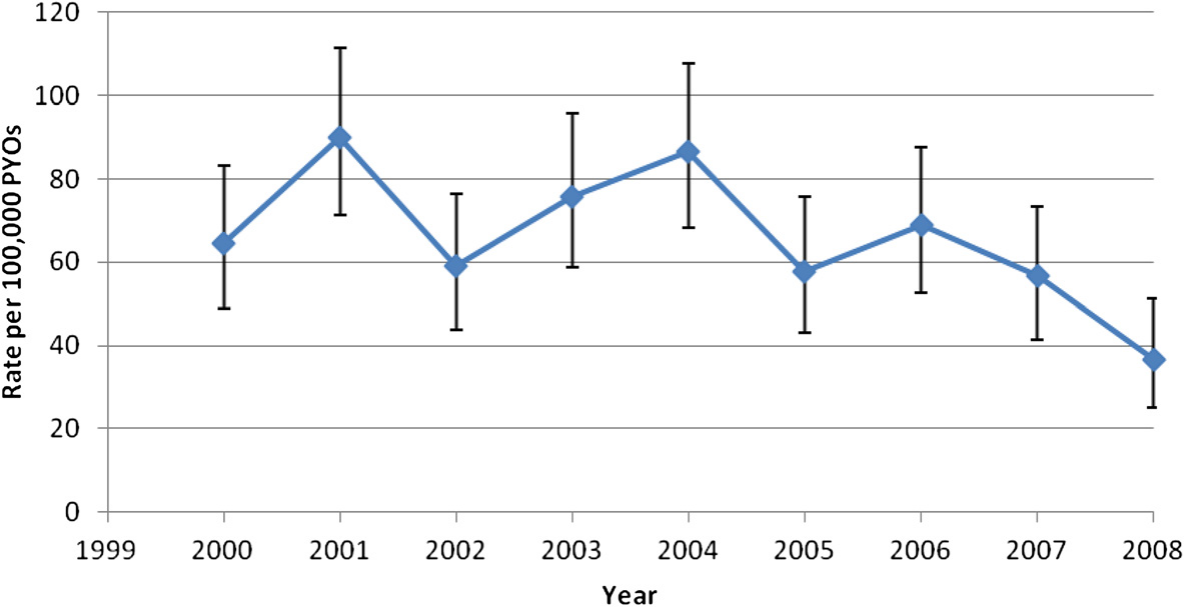
Incidence of homicide trend across a nine-year period (2000–2008)

**Fig. 4:**
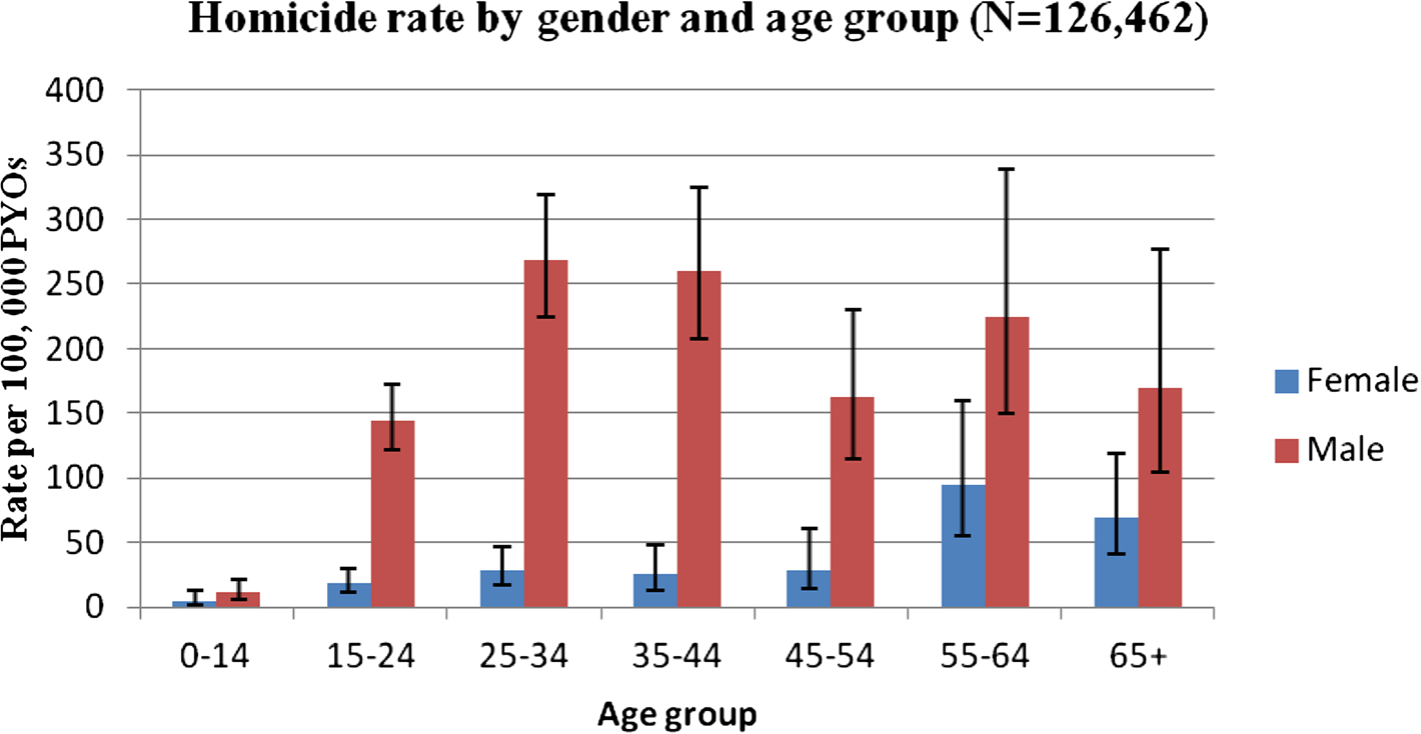
Male and female incidence of homicide by age group (2000–2008)

The overall incidence of homicide was 66 deaths per 100,000 PYOs (95 % CI 60; 72). Male and female rates were 115 (95 % CI 105; 127) and 21 deaths (95 % CI 17; 26) per 100,000 PYOs, respectively. Figure 3 shows a fluctuating homicide rate within the period 2000 to 2006; followed by a suggestion of a decline, which does not show significant trends as shown by the overlapping confidence bands (trend test p-value = 0.340). Homicide rates remain consistently higher among males in all the age groups compared to females (HR = 2.37, 95 % CI; 2.08, 2.70), while it increases by age among females, peaking at age 55–64 with a rate of 76 per 100,000 PYOs (HR = 4.13, 95 % CI; 2.05, 8.31), as shown in Fig. 4.

All the variables with p-values of less or equal to 0.2 in the Weibull univariable regression models were included in the multivariable model (Table 2). Age, sex, residency status, level of education, and socioeconomic status remained statistically significant in the multivariable analysis. Interactions were checked between education and socio-economic status, employment and education, and age and sex, however, only the sex and age interaction was significance (age-sex; p <0.001).

**Table 2:** Stratified multivariable analysis of factors associated with homicide-related death (2000–2008)

	Male (*N* = 59,789)			Female (*N* = 66,670)		
Factors	aHR	95 % CI	P-value	aHR	95 % CI	P-value
Residency						
Resident^a^	1.00					
Partial-resident	0.46	(0.36,0.58)	<0.001	0.34	(0.18,0.63)	<0.001
Nonresident	1.42	(1.03,1.96)	0.034	1.14	(0.54,2.39)	0.730
Education level attained						
None^a^	1.00					
Primary	0.84	(0.57,1.23)	0.376	1.11	(0.56,2.20)	0.749
Post-primary	1.45	(0.98,2.14)	0.059	1.64	(0.78,3.46)	0.188
Socio-economic Status						
Poorest^a^	1.00					
Very poor	1.23	(0.77,1.98)	0.388	0.85	(0.36.1.97)	0.710
Poor	1.19	(0.75,1.89)	0.564	0.72	(0.30.1.71)	0.462
Less poor	1.66	(1.08,2.55)	0.002	0.89	(0.39.1.98)	0.769
Least poor	1.08	(0.68,1.72)	0.736	0.74	(0.30.1.78)	0.503
Age group (years)						
15–24^a^	1.00					
0–14	0.02	(0.01,0.07)	<0.001	0.16	(0.05,0.50)	0.002
25–34	1.67	(1.27,2.21)	<0.001	1.17	(0.59,2.33)	0.650
35–44	1.36	(1.00,1.89)	0.051	0.62	(0.24,1.47)	0.278
45–54	1.07	(0.73,1.53)	0.734	0.96	0.41,2.24)	0.929
55–64	0.07	0.60,1.58)	0.942	3.31	(1.61,6.81)	0.001
65+	0.94	(0.56,1.58)	0.829	2.88	1.38,5.96)	0.004

*aHR* adjusted Hazard Ratio, *CI* confidence interval

^a^Reference category

Factors associated with homicide death among males were place of residence (rural, peri-urban, urban), socioeconomic status, and age group, whereas only place of residence and age were associated with homicide among females. Males and females aged 0–14 years had the lowest risk of homicide in comparison to other age categories. Males aged 25–34 were 17 % more likely to experience homicide (p-value < 0.001) compared to males aged 15–24 years. Females aged 55 years or above had increased risk of homicide death compared to their male counterparts within the same age group. Females’ homicide risk increased with age, peaking in age group 55 to 64 (HR = 3.31, *p* = 0.001).

Although both male and female partial-residents were significantly protected from the risk of homicide compared to residents; the risk was much lower among females than males at 34 % (p <0.001) and 46 % (p < 0.001), respectively. Nonresident males were more than 40 % more likely to experience a homicide death compared to male residents (*p* = 0.001), while there was no evidence of differential homicide deaths between female residents and female nonresidents.

In males there was no clear influence of education on homicide risk. Males within the slightly higher socio-economic position (defined on the basis of assets) were 66 % more likely to die from homicide in comparison to those in the lower socioeconomic status (p-value = 0.002). The same wealth gradient was not evident among females.

**Fig. 5:**
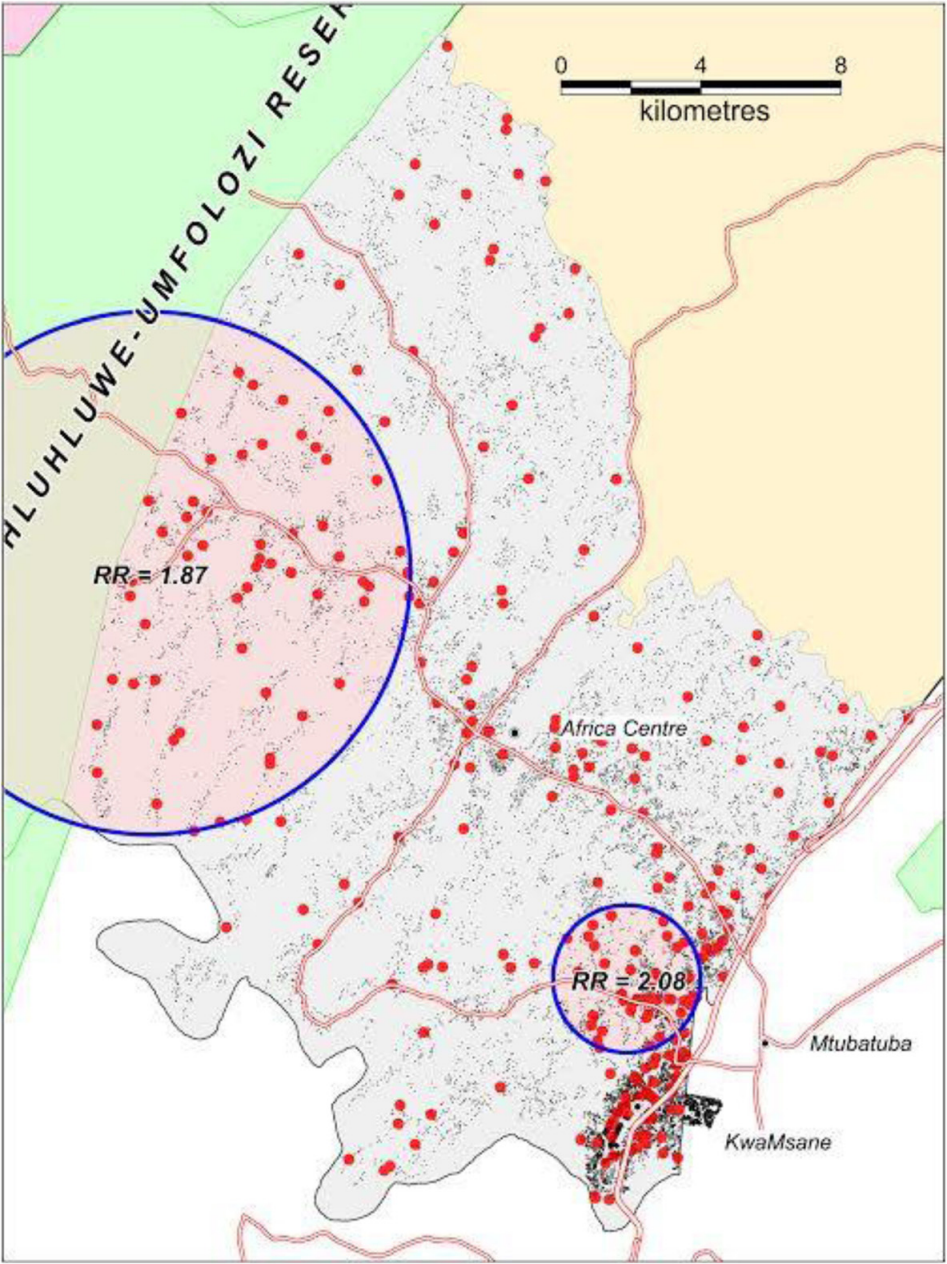
Location of two significant clusters (p < 0.05) with elevated rates of homicide (2000–2008) across the surveillance area. Red dots represent the approximate location of each homicide victim’s place of residence (with intentional random error introduced) and all homesteads (black dots) are depicted

Spatial analysis results revealed considerable geographical variation in homicide rates across the study area. We identified two clusters with an elevated risk (hazard ratio significantly above 1, p–value < 0.05). Cluster 1 (RR =1.87, p-value = 0.04) was in the vicinity of North-Western part of DSA covering the Hluhluwe Umfolozi reserve area (Fig. 5). This area is known to be a hot-spot of factional fighting. Cluster 2 included peri-urban communities along the National Road in the South Eastern part of Africa Centre DSA, (RR = 2.08, p-value = 0.04). This location is characterized by the highest HIV prevalence (Tanser et al, 2009) [25] and incidence (Tanser et al, 2011) in the whole study area [26].

## Discussion

Our study measured a crude homicide incidence rate of 66 deaths per 100,000 person years of follow-up in a rural community in KwaZulu-Natal. This rate is substantially higher than the crude estimate for the whole of South Africa in 2003, when South Africa was second to Columbia at 51 homicides per 100,000 population per year [2, 27]. In 2007–2008, the South African Police Service (SAPS) recorded a homicide rate of 39 per 100,000 populations per year, while NIMSS reported 65 per 100,000 population per year [4]. Suffla et al (2008) reported 85 homicide deaths due to strangulation per 100,000 population per year in Cape Town in 2001 and 67 per 100,000 per year in 2005 [28]. By comparison, a study in Dar es Salaam, Tanzania in 2005 by Outwater et al reported a homicide rate of 13 per 100,000 PYOs [29], while a 2007 injury mortality burden study by Mamady et al in Guinea reported a homicide rate of seven per 100, 000 PYOs [30]. A child homicide mortuary-based study in South Africa by Mathews et al in 2009 documented a homicide rate of 5.5 per 100,000 PYOs for children under the age of 18 years. This was double the World Health Organization’s estimated rate of 2.4 per 100,000 PYOs [31].

Our study is the first study to quantify the levels of and factors associated with homicide-related deaths in a full population cohort in Kwazulu-Natal, and because of its completeness, our findings were not limited by the possibility of bias due to under-reporting. In addition, the spatial statistical techniques employed to identify clustering of homicide cases at the microgeographical scale have never been used before in this type of setting.

Despite the high level of homicide in the study population, the rate is not uniformly geographically distributed. We found two clusters with approximately double the expected risk of homicide, and thus rejected the null hypothesis of no homicide clustering. Clear differences also existed by age and sex, with rates peaking in the age group 25–34 for males and 55–64 for females. This finding was consistent with those of Suffla et al (2008) on female homicidal strangulation in South Africa’s major cities. Suffla’s study found that in three of the major cities, the highest rates of female homicide by strangulation were reported in the over 60 years category, with Durban, the closest major city to the study area, having the highest rate [28]. Previous studies in Tanzania and the United States found similar results [29, 32, 33]. The six-fold difference noted in homicide rate between males and females for the period studied is actually higher than the three-fold difference seen at the global level (men 13.6/100,000; women 4.0/100,000) [1]. The finding conforms to previous studies that have consistently reported uneven distribution of homicide rate by age and sex, with rates substantially lower among females at all age groups, showing that being male is a strong demographic risk factor [6, 34, 35]. More than 90 % of global deaths from injuries occur in low- and middle-income countries. Males in Africa and Central and Latin America have the highest homicide rates in the world [1, 2]. Our findings revealed that the likelihood of an average male falling victim to homicide is strongly influenced by his age, socioeconomic status, and place of residence. In females, only residency and age were significant predictors of homicide risk. Firearm-related fatalities made up a substantial proportion (65 %) of all violent deaths in this study. The finding is consistent with several reports that firearm-related mortality was the major component of homicide in most countries [35–38]. According to United Nations surveys of 69 countries, South Africa has one of the highest firearm-related homicide rates in the world [27, 39]; firearm-related homicide rates in this study are even higher than the national South Africa average (28 per 100,000) [40]. Estimates suggest that there are 11 to 13 million firearms in South Africa, of which 4 million are illegally owned [37].

South Africa’s unique political history and the resulting social and economic inequalities have been identified as some of the possible contributing factors to the high rate of interpersonal violence [5, 37]. Several other factors reported to be associated with violent deaths include poverty, lack of education, unemployment, alcohol abuse, substance abuse, and power (male dominance) [4, 6, 8, 41]. Alcohol is an important contributing factor in fatal and nonfatal injuries in South Africa [42]. Andreuccetti and colleagues, for example, noted that history of alcohol use by victims was greater for homicides occurring over the weekend (56.4 % higher compared to weekdays) [38]. Given the fact that most homicides occurred during weekends and on Fridays in our study, increased alcohol consumption is a plausible explanation for this finding.

In contrast to previous work [4, 43], our study did not find any evidence that poverty is a major predictor of homicide in this population. In fact, among males, belonging to a relatively higher wealth group was associated with a higher likelihood of being a homicide victim. The low survival observed among males in the higher socioeconomic status could imply that wealth increases one’s risk of homicide, especially those considered to be in the relatively higher socio-economic position (less poor category) who are perceived to be economically better off and may be a target of assault, robbery, and subsequently murder. The finding supports Schneider and colleagues’ view that although being in a lower socioeconomic position predicts homicide [44], there is no direct causal relationship between poverty and homicide. However, these results are different from a study on the experiences of violence and socioeconomic position in South Africa, which found that being in the wealthiest quintile was associated with a decrease in risk of violent death [45].

## Conclusion

The high rates of homicide observed in this typical rural South African population – particularly among men – underscore the need for urgent interventions to reduce this tragic and theoretically preventable loss of life in this population and in similar South African settings.
